# The identification of human aldo-keto reductase AKR7A2 as a novel cytoglobin-binding partner

**DOI:** 10.1186/s11658-016-0026-9

**Published:** 2016-10-24

**Authors:** Xin Li, Shanshan Zou, Zhen Li, Gaotai Cai, Bohong Chen, Ping Wang, Wenqi Dong

**Affiliations:** Department of Biopharmaceutics, School of Laboratory Medicine and Biotechnology, Southern Medical University, 1838 North Guangzhou Avenue, Guangzhou, 510515 China

**Keywords:** CYGB, AKR7A2, Protein-protein interactions, Yeast two-hybrid assay, Co-immunoprecipitation, 2-DE, Oxidative stress

## Abstract

Cytoglobin (CYGB), a member of the globin family, is thought to protect cells from reactive oxygen and nitrogen species and deal with hypoxic conditions and oxidative stress. However, its molecular mechanisms of action are not clearly understood. Through immunoprecipitation combined with a two-dimensional electrophoresis–mass spectrometry assay, we identified a CYGB interactor: aldo-keto reductase family 7 member A2 (AKR7A2). The interaction was further confirmed using yeast two-hybrid and co-immunoprecipitation assays. Our results show that AKR7A2 physically interacts with CYGB.

## Introduction

Cytoglobin (CYGB), which is a member of the globin family, was discovered more than a decade ago in a proteomic screen of fibrotic liver [1]. It was originally named STAP (stellate activating protein). Human CYGB is a 190-amino acid, 21-kDa protein [2], encoded by a single copy gene mapped at the 17q25.3 chromosomal segment [3]. It has a compact helical conformation, giving it the ability to bind to heme, which allows reversible binding of gaseous, diatomic molecules, including oxygen (O_2_), nitric oxide (NO) and carbon monoxide (CO), just like hemoglobin (Hb), myoglobin (Mb) and neuroglobin (Ngb) [4]. Unlike those family members, which are respectively localized in the erythrocytes, muscles and neurons and neuroendocrine tissue, CYGB is expressed across a broad spectrum of vertebrate organs and tissues with varying expression levels. It is found predominantly in the fibroblasts of connective tissue and in fibroblast-related cell lineages, such as chondroblasts, osteoblasts, hepatic stellate cells (HSCs) and myofibroblasts, and it may play a role in fibrotic organ disorder [3, 5].

Given its similarity to other globins (particularly Mb and Ngb), several respiratory and stress-related activities for CYGB have been considered. These include oxygen storage, transport and sensing, terminal oxidase activity, nitric oxide dioxygenase activity [6], and scavenging of reactive oxygen species (ROS) [7]. It is also thought to protect cells from reactive oxygen and nitrogen species and deal with hypoxic conditions and oxidative stress in the cells [8]. CYGB is associated with various diseases, such as organ fibrosis in the liver, kidney and pancreas, glaucoma, gastroesophageal reflux disease, and several neurodegenerative disorders. Moreover, considerable evidence suggests that CYGB may function as a tumor suppressor [9].

There has been no definite evidence to support the participation of CYGB in any protein–protein interactions. Although previous studies using the yeast two-hybrid assay and affinity capture discovered that ATPase type 13A2 (ATP13A2) DNA damage-inducible 1 homolog 1 (DDI1) and SH3-domain kinase binding protein 1 (SH3KBP1) were candidate genes for interaction with CYGB, there was no further evidence to support this hypothesis.

In this study, we focused our investigation on possible novel binding partners interacting with CYGB. We used immunoprecipitation combined with a two-dimensional electrophoresis–mass spectrometry assay. Since CYGB has a high expression level in hepatic stellate cells (HSCs) and may play a role in fibrotic organ disorder, we choose LX-2 cells to screen the CYGB-interacting proteins. The putative interactor was further confirmed using the yeast strain Y2HGold in a yeast two-hybrid assay and using mammalian cells in a co-immunoprecipitation assay.

## Materials and methods

### Plasmid constructions

The full-length cDNA of CYGB was inserted into pcDNA3.0-FLAG and pGBKT7 to respectively generate pcDNA3.0-FLAG-CYGB and pGBKT7-CYGB. The full-length cDNA of AKR7A2 was inserted into pCMV-MYC and pGADT7 to respectively generate pCMV-MYC-AKR7A2 and pGADT7-AKR7A2. All of the cDNA was obtained from human hepatic stellate cells (LX-2) via RT-PCR. The primers are listed in Table 1. All constructs were confirmed via sequence analysis.

**Table 1:** Primers used in this study

Constructs	Primer sequence (5′-3′)
pcDNA3.0-FLAG-CYGB	F: AGCTTAGATTACAAGGATGACGACGATAAGATGGAGAAAG
	R: CGGAATTCCTACGGCCCCGAAGAGG
pGBKT7-CYGB	F: CGGAATTCATGGAGACAGGCGAG
	R: AAAACTGCAGCTACGGCCCCGAAGAGG
pCMV-MYC-AKR7A2	F: CGAATTCAAATGCTGAGTGCCGCGTCT
	R: CCGACTCGAGCTAGCGGAAGTAGTTGGGA
pGADT7-AKR7A2	F: CGAATTCATGCTGAGTGCCGCGTCT
	R: CCGACTCGAGCTAGCGGAAGTAGTTGGGA

### Cell culture and transfection

LX-2 cells (donated by Dr. Scott Friedman, School of Mount Sinai, USA) and HEK293T cells (preserved in our laboratory) were both maintained in Dulbecco’s modified Eagle’s medium (Hyclone) containing 10 % fetal bovine serum (Hyclone), 100 units/ml penicillin and 100 μg/ml streptomycin.

One day before transfection, the LX-2 cells were seeded into 6-well culture plates. When the cells reached ~80 % confluence, the pcDNA3.0-FLAG or pcDNA3.0-FLAG-CYGB vector was transfected into the cells using Lipofectamine 2000 (Invitrogen) according to the manufacturer’s protocol. HEK293T cells were co-transfected using pCMV-MYC-AKR7A2 together with pcDNA3.0-FLAG or pcDNA3.0-FLAG -CYGB vector. All cells were cultured at 37 °C with 5 % CO_2_.

### Immunoprecipitation

Whole cell extracts of transfected LX-2 cells were prepared 48 h post-transient transfection in a mild lysis buffer consisting of 20 mM Tris, 150 mM NaCl, 2 % glycerol, 1.6 mM EDTA, 0.5 % Triton X-100 and a protease inhibitor cocktail. The supernatant was collected, incubated with ANTI-FLAG M2 Affinity Gel (Sigma) overnight at 4 °C and then centrifuged for 30 s at 7500 g. The precipitates were washed with 1 ml IP washing buffer A (20 mM Tris, 150 mM NaCl) three times and 1 ml IP washing buffer B (20 mM Tris) twice. Finally, the precipitates were eluted with 300 μl IP elution buffer (6 M urea, 2 M thiourea, 4 % CHAPS, 0.3 % ampholytes, 0.002 % bromophenol blue), and the eluted proteins were prepared for two-dimensional electrophoresis.

### Two-dimensional gel electrophoresis and mass spectrometry

Two-dimensional gel electrophoresis (2-DE) was performed according to the Bio-Rad manual, with the first dimension isoelectrofocusing carried out on 17-cm IPG strips (pH3-10 NL, Bio-Rad) and the second dimension separated by 10 % SDS-PAGE. Before the first dimension isoelectrofocusing, the protein samples were applied to IPG strips for 2 h passive rehydration and 12 h active rehydration loading at 50 V and 20 °C. The first dimension isoelectrofocusing was carried out using Protean IEF Cell (Bio-Rad) with six steps: 250 V for 1 h, 500 V for 1 h, 1000 V for 1 h and 10000 V for 3 h, then 10000 V at 68000 Vhour, and finally 500 V for 3 h. All steps were at 20 °C. After isoelectrofocusing, the proteins were in-gel equilibrated in two steps: with a 50 mM Tris buffer containing 6 M urea, 2 % SDS (*w/v*), 10 % glycerol (*v/v*) and 2.5 % DTT (*w/v*) for 15 min; and with a 50 mM Tris buffer containing 6 M urea, 2 % SDS (*w/v*), 10 % glycerol (*v/v*) and 3.3 % iodoacetamide (*w/v*) for 15 min. The equilibrated IPG strips were then transferred onto two 10 % polyacrylamide slab gels and the SDS-PAGE was carried out in two steps: 10 mA/gel/17 cm for 1 h and 28 mA/gel/17 cm for 9 h. Finally, the separated proteins were visualized via silver staining and image analysis was performed using PDQuest 2-D analysis software V8.0.1 (Bio-Rad). In-gel digestion and MALDI-TOF/MS/MS analysis was performed at the Beijing Genomics Institute in Shenzhen, China.

### Yeast two-hybrid assay

The Matchmaker Gold Yeast Two-Hybrid System (Clontech) was used to identify the interaction between CYGB and AKR7A2. Human CYGB cDNA was inserted into the pGBKT7 vector (Clontech) to generate pGBKT7-CYGB as the bait. Human AKR7A2 cDNA was inserted into the pGADT7 vector (Clontech) to generate pGADT7-AKR7A2 as the prey. Using the small-scale transformation procedure given in the *Matchmaker Gold Yeast Two-Hybrid System User Manual*, we co-transformed 100 ng of each of the following pairs of vectors into Y2HGold Competent Cells: Empty pGBKT7 + Empty pGADT7, pGBKT7-CYGB + Empty pGADT7, Empty pGBKT7 + pGADT7-AKR7A2, pGBKT7-CYGB + pGADT7-AKR7A2, pGBKT7-53 + pGADT7-T as a positive control, and pGBKT7-lam + pGADT7-T as a negative control. The transformed Y2HGold Cells were then plated on SD/-Leu/-Trp Agar and SD/-Ade/-His/-Leu/-Trp/X-a-Gal/AbA agar.

### Co-immunoprecipitation

HEK 293 T cells were co-transfected using pCMV-MYC-AKR7A2 together with pcDNA3.0-FLAG or pcDNA3.0-FLAG-CYGB vectors. Following transient transfection for 48 h, the cells were lysed in a mild lysis buffer consisting of 20 mM Tris, 150 mM NaCl, 2 % glycerol, 1.6 mM EDTA, 0.5 % Triton X-100 and a protease inhibitor cocktail. The supernatant was collected and incubated with ANTI-FLAG M2 Affinity Gel (Sigma) overnight at 4 °C, and then centrifuged for 30 s at 7600 g. The precipitates were washed with 600 μl IP washing buffer (20 mM Tris, 150 mM NaCl) four times. After that, the precipitates were eluted with 2 × sample buffer consisting of 125 mM Tris HCl (pH 6.8) with 4 % SDS, 20 % (*v/v*) glycerol and 0.004 % bromphenol blue, and boiled for 3 min. Finally, the samples were centrifuged for 30 s at 7600 g and the supernatant was prepared for loading on SDS-PAGE and immunoblotting.

## Results

### Identification of AKR7A2 as a possible CYGB interactor

**Fig. 1:**
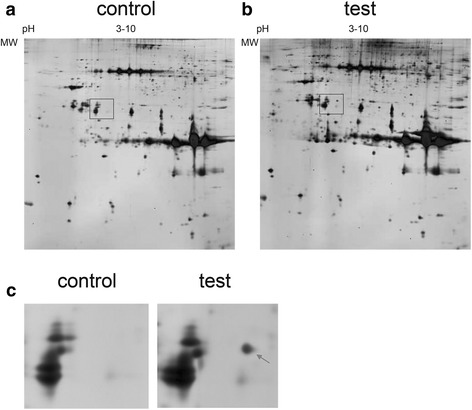
2-DE analysis of differently expressed protein spots. **a** 2-DE image of proteins co-precipitated with FLAG-tag (control). **b** 2-DE image of the proteins co-precipitated with FLAG-tagged CYGB protein (test). The unique spot present in the test but not in the control is in the right half of the area marked with a box. **c** An enlarged view of the area. The arrow indicates the unique spot

**Fig. 2:**
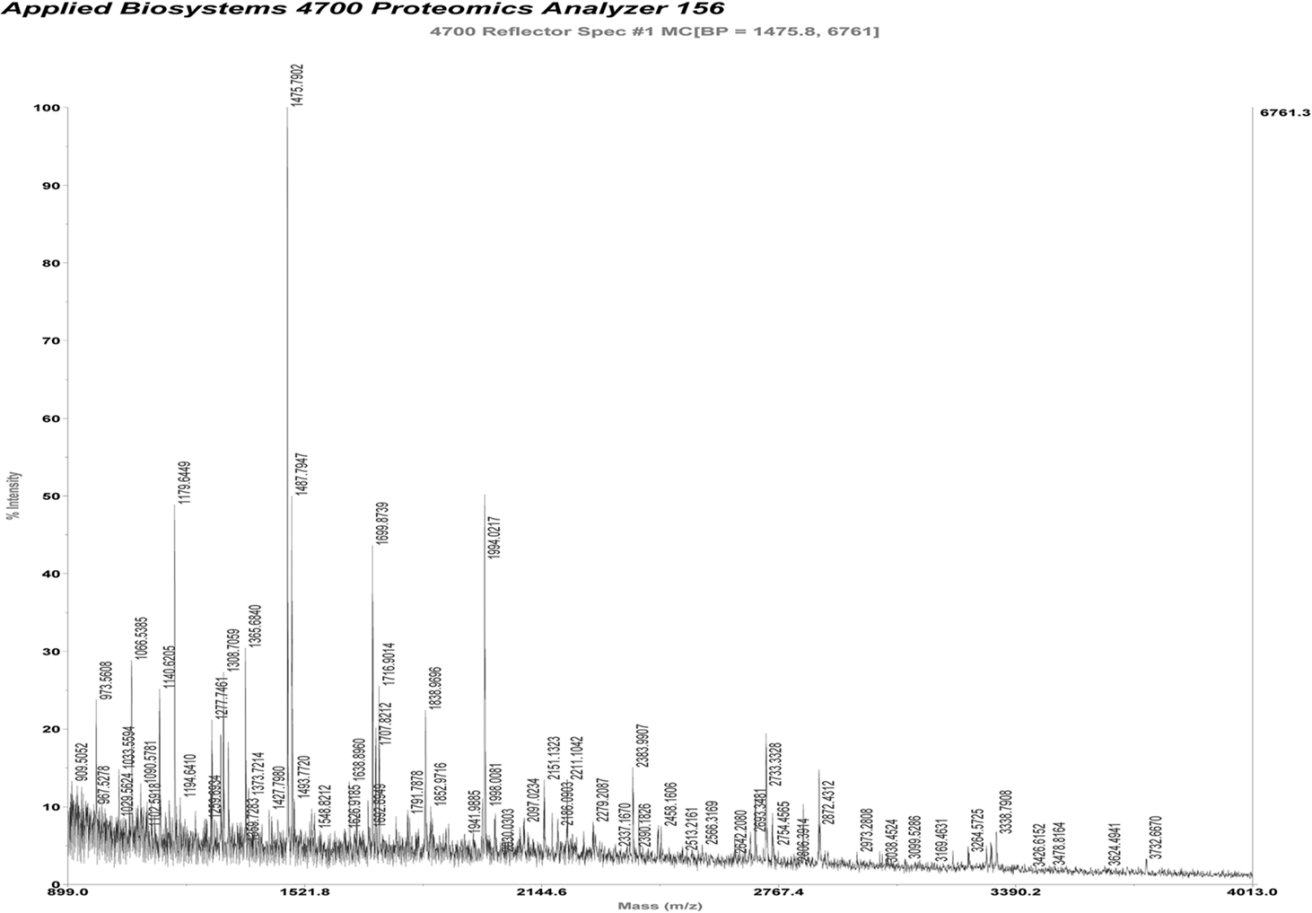
MALDI-TOF/MS/MS results from the analysis of the unique spot in the 2-DE image

In order to identify the interactors of CYGB, we carried out an immunoprecipitation experiment followed by two-dimensional gel electrophoresis (2-DE) and mass spectrometry analysis. FLAG-tagged CYGB protein and FLAG-tag alone were expressed in LX-2 cells and precipitated using ANTI-FLAG M2 Affinity Gel. The proteins co-precipitated with FLAG-tagged CYGB protein or FLAG-tag were used for the 2-DE analysis, the images from which are shown in (Fig. 1). The unique spot present in the experimental gel but not in the control was subjected to in-gel digestion and MALDI-TOF/MS/MS analysis. We then performed a search based on peptide mass fingerprint matching in the Swiss-Prot protein database using the Mascot search engine. Aldo-keto reductase family 7 member a2 (AKR7A2) was identified as a candidate with a significant protein score (*p* < 0.05), and the results of the mass spectrometry are shown in (Table 2) and (Fig. 2).

**Table 2:** Mass spectrometry results of the unique spot in the 2-DE image

Rank	Protein name	Accession no.	Protein score	Protein score C.I. %	Protein MW	Protein PI
1	Aflatoxin B1 aldehyde reductase member 2 (AKR7A2)	sp|O43488|AKR7A2_HUMAN	59	95.568	40019.9	6.7
2	Small ubiquitin-related modifier 1 (SUMO1)	sp|P63165|SUMO1_HUMAN	24	48.644	11606.7	5.35
3	Dedicator of cytokinesis protein 11 (DOCK11)	sp|Q5JSL3|DOCK11_HUMAN	19	2.369	240140.6	7.87

### AKR7A2 interacted with CYGB in the yeast strain Y2HGold

**Fig. 3:**
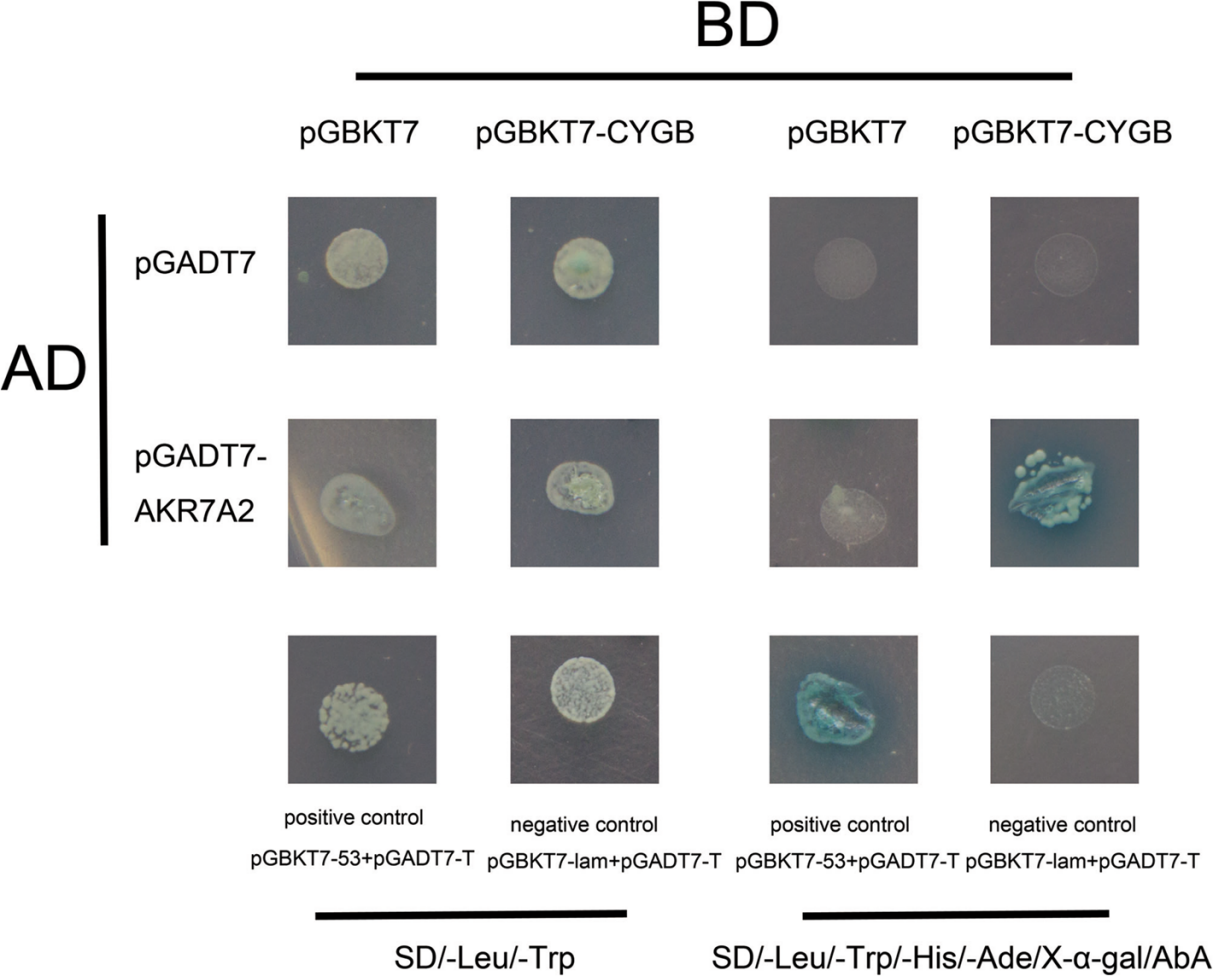
Specificity test of AKR7A2 and CYGB interaction in Y2HGold. Y2HGold cells were co-transformed with Empty pGBKT7 + Empty pGADT7, pGBKT7-CYGB + Empty pGADT7, Empty pGBKT7 + pGADT7-AKR7A2 or pGBKT7-CYGB + pGADT7-AKR7A2 (images as indicated). Positive control: Y2HGold cells transformed with pGBKT7-53 + pGADT7-T. Negative control: Y2HGold cells transformed with pGBKT7-lam + pGADT7-T. The positive interaction between AKR7A2 and CYGB was confirmed by the growth and blue color on SD/-Leu/-Trp/-His/-Ade/X-α-gal/AbA agar plates

To confirm the interaction between AKR7A2 and CYGB, we carried out a yeast two-hybrid assay with Y2HGold competent cells. As shown in (Fig. 3), Y2HGold cells grew on the SD/-Leu/-Trp agar plate, indicating that all the vector pairs were successfully co-transformed into Y2HGold competent cells. On the SD/-Ade/-His/-Leu/-Trp/X-a-Gal/AbA agar plate, only the Y2HGold cells co-transformed with pGBKT7-CYGB, pGADT7-AKR7A2 or the positive control could grow and turn blue, indicating that AKR7A2 interacted with CYGB in Y2HGold.

### AKR7A2 interacted with CYGB in HEK293T cells

**Fig. 4:**
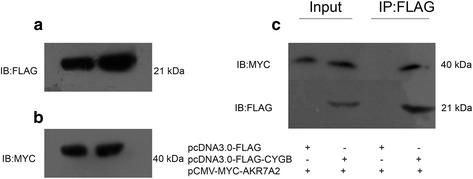
Confirmation of the interaction between AKR7A2 and CYGB in HEK 293 T cells. **a** The expression of FLAG-tagged CYGB protein in HEK 293 T cells. **b** The expression of MYC-tagged AKR7A2 protein in HEK 293 T cells. **c** Co-immunoprecipitation of AKR7A2 and CYGB. HEK293T cells were co-transfected using pCMV-MYC-AKR7A2 together with pcDNA3.0-FLAG or pcDNA3.0-FLAG-CYGB vectors and then subjected to immunoprecipitation with ANTI-FLAG M2 Affinity Gel, followed by immunoblotting with anti-MYC and anti-FLAG antibody

For further verification of the interaction between AKR7A2 and CYGB in mammalian cells, we performed a co-immunoprecipitation experiment. As shown in (Fig. 4a) and (Fig. 4b), FLAG-tagged CYGB protein and MYC-tagged AKR7A2 protein could be stably and highly expressed in HEK 293 T cells, which was significant to the following co-immunoprecipitation experiment. MYC-tagged AKR7A2 protein was detected in the precipitates from cell lysates co-transfected with both proteins (Fig. 4c, lane 4) but not from cell lysates co-transfected with MYC-tagged AKR7A2 protein and FLAG empty vector (Fig. 4c, lane 3). In summary, MYC-tagged AKR7A2 protein was efficiently precipitated by FLAG–CYGB fusion protein but not by FLAG alone, indicating that AKR7A2 interacts with CYGB in mammalian cells.

## Discussion

AKR7A2 is a member of the aldo/keto reductase (AKR) superfamily and AKR7 family. AKRs are a superfamily of NADP(H)-dependent enzymes that reduce aldehydes and ketones to alcohols [10]. Several AKR have been suggested to be under the transcriptional control of nuclear factor erythroid 2-related factor 2 (Nrf2) [11]. The Keap1–Nrf2–ARE pathway is of critical importance in cellular defense against stress, and Nrf2 is reported to mediate the gene expression of AKR7A2 in response to methyl glyoxal (MG) [12]. AKR7A2 is known to be present in a range of tissues, including the liver, kidneys and brain [13]. Previous studies have shown that the AKR7A2 enzyme is catalytically active toward aldehydes arising from lipid peroxidation, suggesting a potential protective role against the consequences of oxidative stress, and representing an important detoxification route in mammals. It may have a role in the defense against ROS, an important factor in disease states that have an oxidative stress component [14], which is similar to CYGB.

Since both AKR7A2 and CYGB are involved in ROS scavenging activity and in the response to oxidative stress, we will explore the possible physiological meaning of this interaction in our future studies.

## Conclusion

In this study, we identified a putative CYGB-interacting protein, AKR7A2, and evidenced its physical interaction with CYGB. These results may provide some clues to the hitherto unknown molecular mechanism of CYGB.

## Abbreviations

2-DE: Two-dimensional gel electrophoresis
AKR: Aldo-keto reductases
AKR7A2: Aldo-keto reductase family 7 Member A2
ARE: Antioxidant response element
ATP13A2: ATPase type 13A2
Cygb: Cytoglobin
DDI1: DNA damage-inducible 1 homolog 1
MG: Methyl glyoxal
Nrf2: Nuclear factor erythroid 2-related factor 2
RNS: Reactive nitrogen species
ROS: Reactive oxygen species
SD: Selection medium
SH3KBP1: SH3-domain kinase binding protein 1
Y2H: Yeast two-hybrid
